# Retraction: AIB1 Cooperates with ERα to Promote Epithelial Mesenchymal Transition in Breast Cancer through SNAI1 Activation

**DOI:** 10.1371/journal.pone.0252791

**Published:** 2021-06-01

**Authors:** The PLOS ONE Editors

Following the publication of this article [1], concerns were raised regarding results presented in Figs 1, 2, 3, 5, 6, and 7. Specifically,

- The WB:α-β-actin panel of Fig 1B appears similar to the WB:α-AIB1 panel of Fig 1E.
- The shAIB1 #1 and shAIB1 #2 panels of Fig 1F partially overlap.
- In Fig 1H vertical irregularities suggestive of gel splicing have been detected between the following lanes:
  ○ WB:α panel lanes 1 and 2
  ○ WB:α panel lanes 2 and 3
  ○ WB:α-E-cadherin lanes 4 and 5
- The following lanes appear similar:
  ○ Fig 1H WB:α-E-cadherin lane 2 (shAIB1 #1) and lane 5 (shAIB1#1+shERα) when flipped horizontally.
  ○ Fig 1H WB:α-β-actin lanes 1–2 (Control and shAIB1 #2) and Fig 3A lanes 5–6 (MCF pdDNA and MCF AIB1) respectively when stretched.
  ○ Fig 1H WB:α-β-actin lanes 4–5 (shERα and shAIB1#1+shERα) and Fig 3A lanes 3–4 (T47D NC and T47D shAIB1#1) respectively when stretched and rotated 180°.
- In Fig 2A the WB:α-ERα lanes 1 and 2 (pcDNA and AIB1) appear similar to the WB:α-β-actin lanes 1 and 2 (pcDNA and AIB1).
- The following panels appear similar:
  ○ Fig 2A pcDNA (0h) and Fig 6C pcDNA 0(h) when flipped horizontally.
  ○ Fig 2A pcDNA (24h) and Fig 6C AIB1 NC (10h) when rotated 180°.
- The Fig 2B NC Migration, Fig 2B shAIB1#1 Migration, and Fig 6D pEGFPC Invasion panels partially overlap.
- The following panels appear similar, despite being used to represent different samples
  ○ Fig 2B AIB1 Invasion and Fig 6D AIB1 NC invasion
  ○ Fig 2B NC Invasion, Fig 2B shAIB1#1 Invasion, and Fig 6D AIB1 shSNAI1 Invasion when flipped vertically.
- The pcDNA α-E-cadherin, shAIB1#1 α-E-cadherin, and shAIB1#1 α-N-cadherin panels of fig 3B partially overlap.
- The following lanes of Fig 5B appear similar despite being used to represent different samples:
  ○ Promoter region A α-AIB1 and α-ERα
  ○ Promoter region B α-AIB1 and α-ERα
  ○ Promoter region C α-AIB1 and α-Erα
- In Fig 7A the α-ERα Invasive front panel appears similar to the α-SNAI1 Invasive front panel, despite being used to represent different samples.

The authors stated that the original data underlying the majority of published results are no longer available due to the time elapsed since the experiments were conducted.

In light of the concerns affecting multiple figure panels that question the integrity of these data, the *PLOS ONE* Editors retract this article.

MW agreed with the retraction but stands by the article’s findings. FZ, SL, AKC, ZJ, YC, FX, HP, and HW either did not respond directly or could not be reached.
